# A South‐to‐South Cultural Adaptation of an Evidence‐Based Parenting Program for Families in the Philippines

**DOI:** 10.1111/famp.12625

**Published:** 2021-01-06

**Authors:** Bernice Landoy Mamauag, Liane Peña Alampay, Jamie M. Lachman, Bernadette J. Madrid, Judy Hutchings, Catherine L. Ward, Frances Gardner

**Affiliations:** ^1^ Department of Psychology Ateneo de Manila University Quezon City Philippines; ^2^ Division of Social Sciences College of Arts and Sciences University of the Philippines Visayas Miagao Iloilo Philippines; ^3^ Centre for Evidence‐Based Intervention, Department of Social Policy and Intervention University of Oxford Oxford UK; ^4^ MRC/CSO Social and Public Health Sciences Unit University of Glasgow Glasgow UK; ^5^ Child Protection Unit, Philippine General Hospital University of the Philippines Manila Manila Philippines; ^6^ School of Psychology, College of Health and Behavioural Sciences, Centre for Evidence Based Early Intervention Bangor University Gwynedd Wales UK; ^7^ Department of Psychology University of Cape Town Rondebosch South Africa

**Keywords:** Cultural Adaptation, Parenting Program, Filipino Parenting, Child Maltreatment

## Abstract

Rates of child maltreatment are higher in low‐ and middle‐income countries due to risk factors such as social inequities, economic adversity, and sociocultural norms. Given the evidence showing the effectiveness of parenting interventions to prevent child maltreatment, this study embarked on a cultural adaptation of an evidence‐based parenting program with the eventual goal of integrating it within a nationwide conditional cash transfer program for low‐income Filipino parents with children aged 2–6 years. We document the systematic adaptation of the Parenting for Lifelong Health for Young Children program that was developed and tested in South Africa, for low‐resource Filipino families using the heuristic framework for the cultural adaptation of interventions. We underscore the merits of conducting a multistage top‐down and bottom‐up process that uses a participatory approach among cultural insiders and outsiders to develop a parenting intervention that reflects the contextual realities and cultural values of end users. The adapted program, *Masayang Pamilya Para sa Batang Pilipino*, is the product of a delicate and deliberate effort to balance Filipino childrearing goals and values with the scientific evidence on components of parenting interventions known to promote positive parenting and prevent child maltreatment.

## Resumen

Los índices de maltrato infantil son más altos en los países de ingresos medios y bajos debido a factores de riesgo, como las desigualdades sociales, las dificultades económicas y las normas socioculturales. Teniendo en cuenta los datos que demuestran la eficacia de las intervenciones en la crianza para prevenir el maltrato infantil, este estudio inició una adaptación cultural de un programa de crianza factual con el objetivo principal de integrarlo dentro de un programa de transferencia condicional de dinero en efectivo a nivel nacional para padres filipinos de bajos recursos con niños de entre dos y seis años. Documentamos la adaptación sistemática del programa “Crianza para una buena salud durante toda la vida” (Parenting for Lifelong Health, PLH) orientado a niños pequeños que se desarrolló y se probó en Sudáfrica, para familias filipinas de bajos recursos utilizado el marco heurístico para las adaptaciones culturales de las intervenciones. Subrayamos los méritos de llevar a cabo un proceso multietapa descendente y ascendente que emplea un método participativo entre personas conocedoras de las culturas y personas ajenas a ella para desarrollar una intervención en la crianza que refleje las realidades contextuales y los valores culturales de los usuarios finales. El programa adaptado, *Masayang Pamilya Para sa Batang Pilipino*, es el producto de un esfuerzo comprometido y deliberado de equilibrar los objetivos y los valores de la crianza de los niños filipinos con las pruebas científicas sobre los componentes de las intervenciones en la crianza que promueven la crianza positiva y previenen el maltrato infantil.

## 摘要

由于社会不平等、经济困境和社会文化规范等风险因素，在低收入和中等收入国家里儿童受虐待的比率较高。鉴于那些能显示通过家长教育干预防止儿童虐待现象这种措施的有效性的证据, 本研究开展了以证据为基础的文化适应教育计划，其最终目的是为了将这种措施整合在一个全国性的有条件的现金转移计划中，对象为菲律宾低收入父母， 家有2 ‐ 6岁儿童。我们记录了在南非开发和测试的儿童终身健康养育(PLH)项目在资源贫乏的菲律宾家庭的适用的情况，该项目使用启发式的框架来适应干预的文化适应性。我们强调，采用由上而下和由下而上的多阶段过程，在文化内部人士和外部人士之间采用参与式方法，以发展一种反映终端用户的环境现实和文化价值的育儿干预，这些做法是有诸多好处的。经过改编的节目Masayang Pamilya Para sa Batang Pilipino是一项细致而深思熟虑的努力的成果，目的是在菲律宾的育婴目标和价值观与促进积极育儿和防止虐待儿童的育儿干预措施组成部分的科学证据两者之间实现平衡。

Policy recommendations to implement interventions for the prevention of violence against children have increased over the years (World Health Organization, 2016). Parenting interventions are systematic programs in which “at least one component of the intervention involves activities designed to promote some aspect of effective parenting” (Sandler, Schoenfelder, Wolchik, & MacKinnon, 2011, p. 302). Such programs promote behavior change in caregivers and expand their repertoire of positive child behavior management skills via processes associated with social learning theory and principles from behavior therapy. These skills, in turn, are maintained by the positive results that they see in their children’s behaviors (Sandler et al., 2011). Evidence of program effectiveness includes reductions in child maltreatment and child disruptive behavior (Chen & Chan, 2015; Leijten et al., 2018).

Parenting interventions are especially important in low‐ and middle‐income countries (LMICs) and other contexts of significant economic deprivation. Parents in poorer communities are at higher risk of maltreating their children (Hillis, Mercy, Amobi, & Kress, 2016; Ward, Sanders, Gardner, Mikton, & Dawes, 2015). Promoting positive forms of parenting may be an efficient and cost‐effective way to address child behavior problems and prevent delinquency, substance misuse, and longer‐term mental health problems (Leijten, et al., 2018; Ward et al., 2015). At the same time, contextually relevant approaches are necessary to address prevailing cultural norms in LMICs, such as the acceptability of corporal punishment (Mejia, Leijten, Lachman, & Parra‐Cardona, 2016).

One potential strategy to establish parenting interventions in LMICs is to culturally adapt evidence‐based parenting principles and/or programs that were developed and tested elsewhere, mostly in high‐income countries (HICs; Baumann, et al., 2015). Given the limited resources in LMICs, transporting an evidence‐based parenting program may be more efficient than building parenting programs from the bottom‐up (Gardner, Montgomery, & Knerr, 2016). When introduced into LMICs, intervention programs may require modifications to content and delivery. Adaptations may also be needed to integrate the program within a specific context to enhance acceptability and effectiveness, while maintaining fidelity to core program components (Castro, Barrera, & Martinez, 2004; Parra‐Cardona et al., 2018).

In this paper, we describe a South‐to‐South cultural adaptation process of an evidence‐based parenting program in the Philippines that was originally developed and tested in South Africa to decrease the risk of child maltreatment and improve child well‐being (Lachman, Sherr, et al., 2016; Ward et al., 2019). We provide a model of a systematic adaptation process for intervention initiatives in other LMICs that seek to balance evidence‐based approaches with local contextual and cultural realities (Parra‐Cardona et al., 2018). We also illustrate how cultural contexts may nuance intervention programs and their delivery, and provide valuable lessons for family interventionists, particularly those whose work is situated in non‐Western contexts. We therefore address the necessity for systematic documentation of adaptation efforts to clarify the core components of interventions that are critical in reproducing expected outcomes in different contexts (Mejia et al., 2016).

## Filipino Parenting as Context for Cultural Adaptation

### Cultural values that shape Filipino parenting

The Filipino family is characteristically cohesive, with members showing filial respect and mutual support for each other. Respect for parents is grounded in the cultural value of *utang na loob* or the immeasurable “debt of one’s being” toward parents for having raised them (Enriquez, 1994). Deference toward parents and elders is codified in the words *po* and *opo* when addressing them (Guthrie & Jacobs, 1966), and in expectations that children submit to authority and meet obligations to support the family as they grow older (Alampay & Rothenberg, in press).

Children’s behaviors, achievements, or shortcomings reflect on the family. The cultural value of *hiya* or a sense of propriety means that one should consider family in decisions and actions (Enriquez, 1994). Subtle and often nonverbal dynamics of interpersonal interaction known as *pakikiramdam*, or “being sensitive to and feeling one’s way toward another person” (Mataragnon, 1988, p. 252), are intended to protect the self and family from *kahihiyan* (shame). Accordingly, humility and modesty (Choi, Park, Lee, Kim, & Tan, 2017), and emotional control (Ng & Wang, 2019), are qualities valued and inculcated by Filipino parents.

### Filipino childrearing attitudes and behaviors

In Filipino and other Asian families, instrumental forms of support are more typical than overt demonstrations of warmth (e.g., praise; Ng & Wang, 2019). For instance, Filipino parents prepare a favorite meal, or provide school needs despite financial hardships, to signify positive regard (Alampay & Rothenberg, in press). Filipino parents have also been found to hold traditional and authoritarian attitudes toward childrearing, valuing obedience more than self‐direction (Alampay & Jocson, 2011). Such authoritarian attitudes are positively associated with the endorsement and use of corporal punishment, with years of education inversely associated with these attitudes and behaviors (Jocson, Alampay, & Lansford, 2012).

Evidence of the pervasive use of corporal punishment by Filipino parents and cultural attitudes and beliefs that support corporal punishment were important contexts to our adaptation of an evidence‐based parenting program in the Philippines. In a national violence against children study involving approximately 4,000 Filipino youth aged 13–24, one in two participants reported having experienced mild forms of physical violence (such as spanking using bare hands) and a third have experienced psychological violence (Council for the Welfare of Children & UNICEF Philippines, 2016). A third also reported having experienced severe forms such as being slapped or kicked. Mothers were most frequently identified as using corporal punishment, likely because they are most engaged in caregiving, followed by fathers, older brothers, and older sisters.

## The Parenting for Lifelong Health for Young Children Program

This study is part of an international initiative called Parenting for Lifelong Health (PLH) that aims to develop, test, and disseminate a suite of low‐cost parenting programs to prevent child maltreatment and improve parent and child well‐being in LMICs.^1^ This paper reports on the cultural adaptation for the Philippines of PLH for Young Children (PLH‐YC), a group‐based parenting program for caregivers of children aged two to nine years. PLH‐YC was developed and evaluated in Cape Town, South Africa, and incorporates the principles and components of parenting interventions grounded in social learning theory and child behavior management techniques (Hutchings, 2013; Lachman, Sherr, et al., 2016). PLH‐YC was specifically designed to be low cost with a participatory delivery approach applied by trained paraprofessionals. Facilitators follow a structured, manualized curriculum based on a theory of change model derived from the works of Forehand and McMahon (2003) and Hanf (1969) that emphasize the importance of establishing a strong foundation of positive parent–child relationships, prior to learning behavior management and discipline strategies. Results from a randomized controlled trial (RCT) in Cape Town, South Africa, found parent‐reported increases in positive parenting and decreases in harsh parenting immediately after the program, and significantly fewer observed negative parenting strategies one year after program completion (*N* = 296; Ward et al., 2019).

The objective of this current adaptation study was to examine (1) the preferences and perceptions of Filipino parents and service providers regarding content for parenting programs in the Philippines and (2) the cultural and contextual relevance and acceptability of the adapted program for very low‐income families with children aged 2–6 years,^2^ for eventual delivery within a nationwide conditional cash transfer system. We applied the heuristic framework for the cultural adaptation of interventions by Barrera and colleagues (Barrera, Castro, Strycker, & Toobert, 2013). This stage model integrates top‐down and bottom‐up approaches to cultural adaptation, wherein we begin with an existing PLH‐YC program and consider its theoretical underpinnings and the evidence of good practices (top‐down). Next, members of the recipient cultural group (i.e., target families, program facilitators, and researchers) subject the program to careful examination and provide feedback at various stages (bottom‐up). The top‐down and bottom‐up processes interact in an iterative fashion. The framework unfolds in a series of steps that include Phase 1: Information gathering, Phase 2: Preliminary adaptation design, Phase 3: Preliminary adaptation tests, and Phase 4: Adaptation refinement. Information gathering entails the collection of data from the target recipients of the program, which then informs the preliminary adaptation design. The adapted program is then pilot‐tested on a small scale (Phase 3), and the results inform another round of adaptation on the content and delivery of the program (Phase 4). In this paper, we describe the adaptation process and resulting changes that were made to PLH‐YC to make it acceptable and relevant to low‐income Filipino parents of young children.

## Methods

### Procedures and Participants

Study protocols were reviewed and approved by the ethics review boards of the Ateneo de Manila University (AdMUREC_16_014PA) and the Universities of Oxford (R43041/RE002) and Cape Town (PSY2016‐041). All participants were recruited using purposive sampling according to the following criteria: (1) parents or caregivers of children aged 2–6 years; (2) recipients of the Philippine government’s conditional cash transfer (CCT) program (Phase 1 only); and (3) willing and able to take part in a weekly, 12‐session, group‐based parenting program (Phase 3 only). All participants underwent an informed consent process and signed informed consent forms prior to participating in the study.

#### Phase 1: Information gathering

We conducted interviews and focus group discussions (FGDs) with parent beneficiaries of the national CCT program to examine the contents Filipino parents consider important in a parenting program. The CCT program provides cash benefits to low‐income families amounting to $10 per month and $6 per child, subject to their compliance with health, education, and other social development conditions (including attendance to a parenting program). Twenty‐seven mothers and 20 fathers, representing 47 families from one urban poor community in Metro Manila, participated in five FGDs. Mean age of children was 4.52 (*SD* = 1.69), and majority of participants were high school graduates.

#### Phase 2: Preliminary adaptation design

We examined results from Phase 1 to inform the initial adaptation of the program. The program design team, comprised of the Filipino research team and international collaborators (i.e., the authors of this paper), participated in an intensive workshop in the Philippines and held several online meetings to discuss the adaptation of the program vis‐à‐vis the data. We examined the original program content, structure, materials, and modes of delivery to identify components that were compatible or incompatible with local features and practices, and which conflicted with or supported Filipino parents’ needs, values, and beliefs. These components ranged from the characters and scenarios depicted in materials, to more fundamental messages in the program content. Based on Phase 1 data and local collaborators’ emic perspectives, specific recommendations were discussed to make the program appropriate and meaningful to Filipino parents. The team documented their adaptations in drafts of the facilitator guides and session manuals developed in‐person and online as a collaboration between the local and international team members.

#### Phase 3: Preliminary adaptation tests

The adapted program was tested in a feasibility pilot study to assess participants’ perceptions of the relevance and acceptability of specific content and delivery approaches. Participants included 29 mothers and one father (*M* age = 33.30, SD = 10.54) who were all caregivers of children aged 2–6 years (*M* age = 3.87, *SD* = 1.33). The demographic characteristics and poverty indicators of the sample are presented in Table 1. Caregivers attended one of two parent groups, each led by a pair of Filipino facilitators who were trained by the third author. The facilitators comprised a psychology graduate student, a physician, and two social workers.

**Table 1:** Sociodemographic Characteristics of Pilot Test Participants (*N* = 30)

Number of people in household, *M* (*SD*)	7.67 (3.71)
Number of children in household, *M* (*SD*)	3.10 (1.77)
Parent completed high school, *n* (%)	12 (40%)
Parent is unemployed, *n* (%)	20 (66.7%)
Household assets (min of 4 max of 10 in the sample)^a^, *M* (SD)	5.97 (1.59)
Experience of household hunger ≥5 times in previous month^b^, *n* (%)	10 (33.4%)
Parent experienced violence as a child^c^, *n* (%)	22 (73.3%)
Parent experienced intimate partner violence^d^ in previous month, *n* (%)	10 (33.3%)

^a^ Economic Asset Index; adult‐reported access and ownership of goods such as water, electricity, mobile phone, and TV.

^b^ Hunger Scale Questionnaire (Labadarios, 2003); adult‐reported occurrence of hunger in household.

^c^ ICAST‐Retrospective (Dunne et al., 2009); adult‐reported incidence of childhood physical abuse, emotional abuse, and neglect.

^d^ Revised Conflict Tactics Scale Short Form (Straus et al., 1996); adult‐reported incidence of physical and psychological intimate partner violence.

The parent groups met once a week in the *barangay* (village) center. Apart from one dropout, all parents participated in at least one session (96.6% enrollment). The overall mean attendance rate was 62% or 7 out of 12 sessions. Absences were spread throughout the 12 sessions of the program, and participants who missed a session were encouraged by facilitators to attend the succeeding sessions. Following program completion, two FGDs were conducted with 19 program participants (*n* = 10 in the first FGD, *n* = 9 in the second FGD) and another with three of the four program facilitators to examine the relevance and appropriateness of the intervention components.

#### Phase 4: Adaptation refinement

We used the data from the FGDs to further refine the program. Two of the international collaborators (JML and JH) came to the Philippines to participate in two days of intensive meetings with the local collaborators and facilitators to discuss the importance and relevance of program content, challenges and good practices in delivery, and other recommended improvements to the adapted program.

### Data Analysis

Prior to data analysis, the first author and three research assistants transcribed the audio recordings and conducted accuracy checks. This allowed them to immerse themselves in the data to establish trustworthiness in the research process (Morrow, 2005). They then analyzed the FGD transcripts following the six phases of thematic analysis by Braun and Clarke (2006). The researchers first familiarized themselves with the data by reading and rereading the transcripts and noting their interpretations. Each researcher then combed through the data set to code and identify patterns as potential themes. They collectively discussed their individual work to determine consistent and contrasting patterns. The latter were addressed via discussions on the latent meanings and contents of themes until consensus was attained. The researchers then applied the group‐derived codes and themes to the data set. Finally, they defined and finalized theme labels, and selected coded data extracts to substantiate these themes.

Validity checks were conducted through consultations with the authors who corroborated or proposed alternative interpretations to the coded data. The ongoing dialogue with local and international coresearchers was a reflexive strategy that allowed beliefs, perspectives, and biases to be checked and supports the trustworthiness of the research process and findings (Morrow, 2005). Reliability checks were done via thorough documentation of the analysis procedure, meeting notes, and drafts of outputs.

## Results and Discussion

### Phase 1: Information Gathering

Five themes emerged from interviews and FGDs regarding content that parents considered important to include in a parenting program: (1) promoting positive interactions with children, (2) enabling children to express emotions, (3) communicating effectively with children according to their developmental level, (4) strengthening desirable positive social behaviors, and (5) effective discipline and managing difficult child behavior.

#### Promoting positive interactions with children

Participants alluded to the need to have more positive interactions with their children in order to strengthen their relationship. As one mother articulated:

I think it is essential so that you don’t lose your children’s affection. It is important that while they’re growing up, their relationships with their parents get better and better.

These positive interactions were viewed as necessary to improve compliance, maintain harmonious relationships, and overcome daily child behavior challenges. This also suggests Filipino parents’ openness to apply more nurturing and affectionate approaches to parenting rather than the authoritarian practices described in the literature.

#### Enabling children to express emotions

Participants wanted to learn how to assist their children to express emotions. A mother explained that young children often find it difficult to express their emotions constructively, especially if these are negative. It has been documented in a seminal qualitative study that the expression of aggression or anger is discouraged in Filipino culture (Guthrie & Jacobs, 1966) as is the case in other Asian contexts (Ng & Wang, 2019). Parents added that teaching children how to express emotions was necessary so that their children would understand their own and their parents’ emotions, which is a critical aspect of *pakikiramdam* (sensitivity to others) and relating harmoniously with others.

#### Communicating effectively with children according to their developmental level

Participants identified the importance of knowing how to interact with their children in accordance with the child’s age. One father articulated that “It's important that parents know what to say to their children so that they wouldn't get involved in those kinds of things [risk‐taking behaviors].” Fathers also explained that this topic would help them to learn how to encourage their children to be more open with them, especially since Filipino fathers are less involved in the daily care of children (Jocson et al., 2012). A mother shared that she did not know how to instruct her young daughter and this resulted in misunderstandings. The skill of communicating with children more effectively will enable parents to guide children toward expected behaviors, support compliance, and improve parent–child relationships.

#### Strengthening desirable positive social behaviors

Participants wanted to know how to maintain and strengthen desirable social behaviors in children, especially obedience and respect for elders. One mother reported how these behaviors were a reflection on themselves.

I think it's important that children learn these things [how to behave in public] because it's a reflection of your parenting. Their behavior around others would reflect you as a parent. There are children who use ‘po’ or ‘opo.’ The parents of these children get praised. People would say ‘Your mother is excellent’.

This goal supports the prevalent tendency among Filipinos to maintain good relations with others and avoid familial shame (Enriquez, 1994).

#### Effective discipline and managing difficult child behavior

Participants disclosed that they spanked using their bare hands or used implements in order to discipline. Aside from spanking, parents disciplined by talking to their children, pinching, scaring, and using verbal punishment. At the same time, respondents revealed that these practices were not necessarily effective because their children’s misbehaviors continued, and thus expressed a desire to learn more effective ways of disciplining:

Sometimes, when we talk to our child and he doesn't listen, he gets spanked. There might be other ways of disciplining a child who doesn't straighten up when spoken with.

This sentiment is consistent with the worldwide data on high rates of physical punishment in the home despite parents expressing the view in surveys that corporal punishment is not necessary or desirable (UNICEF, 2010).

### Phase 2: Preliminary Adaptation Design

The authors analyzed the themes of participants’ responses in Phase 1 vis‐a‐vis the original PLH‐YC content in order to adapt the program for Filipino families. Our adaptations range from *surface* to *deep* (Resnicow, Soler, Braithwaite, Ahluwalia, & Butler, 2000). Surface adaptations refer to fitting the program with the target population’s observable features and behaviors, such as language translation and changing the appearance of characters in materials. Deep adaptations address sociocultural, historical, and psychological aspects that influence behaviors differently in the local context. This involved reframing messages to suit Filipino parents’ values and motivations.

The themes largely corresponded with the content of the original program, and many of the concerns, desired program content, and values described by Filipino parents were similar to those identified by South African parents (see Lachman, Sherr, et al., 2016). As a result, the social learning and participatory problem‐solving principles of PLH‐YC were maintained in the adapted program, as well as the core content and parent skills in each session. The adapted program likewise aimed to decrease the risk of child maltreatment, enhance parents’ skills to manage challenging child behavior using nonviolent strategies, reduce child behavior problems, and increase caregiver and child well‐being.

#### Program content

We renamed the adapted program *Masayang Pamilya Para sa Batang Pilipino* (or Happy Family for the Filipino Child), with the abbreviation, *MaPa*, which signifies “map” in Filipino, as well as shorthand for mother and father. The focus on a positive emotion (*masaya* or happy) and on the whole family is aimed to reduce stigma and *hiya* (shame) that Filipino parents may feel if a program is labeled as violence prevention for parents. The two‐stage Hanf model (1969) was represented in an image of a typical Filipino house or *Masayang Tahanan* (Happy Home), adapted from the *rondavel* or thatched hut that is used as a visual framework in PLH‐YC (Figure S1). Parents are coached to strengthen the walls of their house, representing the foundation of positive parenting, before focusing on the roof which symbolizes limit setting and effective nonviolent discipline. The visual framework is delivered over 12 group sessions, each focusing on a specific core theme or skill (Table 2). The metaphor of a house and core skills as “building blocks” allow parents to easily grasp that they are progressively developing skills to build a happy home.

**Table 2:** Masayang Pamilya Para sa Batang Pilipino Program Sessions

Session	Title/Theme	Key Parent Learning Outcomes
1	One‐on‐one time with your child	Engage in focused, child‐directed interactions within specific and regular times (e.g., 10 minutes every day)
2	Say what you see	Observe and verbally describe the child’s behaviors or other aspects of the environment to the child
3	Talking about feelings	Notice and communicate about one’s own and child’s positive and negative feelings
4	Praising our children	Give specific praise that is contingent on the child’s positive behavior
5	Simple rewards	Provide a tangible, practical, culturally acceptable reward for the child’s achievement of specific behavior goals
6	Giving positive instructions	Communicate positively stated (i.e., the desired behavior), specific, and realistic instructions to the child
7	Establishing household rules and routines	Establish positively stated, specific, and realistic household rules (e.g., study time, chores) and routines especially mealtimes and bedtimes
8	Redirecting negative behaviors	Use redirect and ignore to address emerging child misbehavior and negative attention‐seeking behavior
9	Cool down for children	Implement time‐out for dysregulated child behavior
10	Using consequences	Establish and implement consequences for child’s noncompliance to rules and instructions
11	Avoiding and resolving conflicts	Communicate about and problem‐solve conflicts with the child’s involvement
12	Reflection, celebration, and moving on	Plan how to sustain behavior change and provide support for each other in the parent group after the program

Note

Mindfulness and stress‐reduction activities (e.g., physical exercises, brief meditations) were included in all sessions.

#### Program delivery approach

The intervention approach is participatory and nondidactic. The program begins with individual consultations, usually at the home of the parent, during which facilitators help parents identify specific behavior goals for themselves and/or their children. In the program sessions, facilitators support skills acquisition by helping parents identify parenting principles and strategies using illustrated stories, and then coach parents as they practice these skills in role‐plays. Parents are given activities to practice with children at home. Text/SMS messages are sent by facilitators to parents some days after each session to encourage them to practice the learned skills. Home visits are conducted with parents who miss sessions or need additional support in problem‐solving the challenges that emerge from practicing skills with the child.

Illustrated stories, referred to as *komiks*, represent a key strategy for engaging parents in a simple and culturally significant way, as *komiks* are popular media in Filipino culture (Reyes, 1997). The stories were adapted to incorporate characters, topics, and scenarios relevant to Filipino family and community life. All of the *komiks* depict the experiences of two Filipino families as described by participants in Phase 1: one family living in an informal settlement in the city and composed of a grandmother, a mother, and two children (the father is a migrant worker who comes home occasionally); the other family living in a rural *barangay* (village) and composed of a father, a mother, and two children. Certain scenarios from the original program were modified according to contextual realities. For example, playing dress‐up in child‐directed play was changed to an *adobo‐*cooking pretend play, as dress‐up is less typical and appropriate in the low‐income setting (see Figure S2).

#### Specific contextual adaptations

A number of deeper adaptations were made to better align the content with Filipino values and practices and increase the cultural acceptability of the program. These adaptations were explicitly highlighted in the facilitator training and manual. Given traditional attitudes and practices that emphasize authority among Filipino parents, the team expected the concept of “child‐directed” activities, the core lesson of Session 1, to be challenging. Hence, we carefully framed this concept to emphasize that it is a specific, time‐bound period for parents to allow children to take responsibility for an activity, rather than the parent relinquishing control to the child. In addition, benefits of child‐directed activity were to be elicited from parents and thoroughly discussed in the session.

Similar to the South African context, we noted that praising children was not a normative practice in Filipino households. Consequently, the facilitators were taught to elicit the specific words or terms from parents themselves which they would feel comfortable using to express their approval of their children’s behaviors. These words are then practised in role‐plays during sessions and at home, and in session activities among parents so as to normalize and validate praising. We also framed content on rewards toward encouraging parents to think about and apply rewards that are practical, realistic, and culturally acceptable (e.g., the child’s favorite dish, extra time for play).

Communicating about feelings was nuanced to emphasize the acceptability of expressing negative emotions in an appropriate way. Facilitators were reminded to elicit from parents the benefits of allowing children to express and label emotions, and to explore barriers to children’s expression of emotions (i.e., that this may be seen as defiance of parental authority or disobedience).

To address parents’ desire to have content on ways to communicate with children according to the child’s developmental level, additional discussion questions were incorporated on the significance of clear and positive instructions, and household rules and routines for young children (e.g., why and how complex instructions are broken down to discrete steps). A new section on developmental milestones for young children was added to the manual to prepare facilitators to address concerns about age appropriateness of expectations that can arise across all the sessions.

The deliberate omission of messages that explicitly disapprove of, or discourage, the use of corporal punishment was maintained from the original program. Corporal punishment is a normative practice in many families, and labeling it as negative or prohibiting its use may be deemed culturally insensitive and could result in parents feeling disrespected and disempowered. MaPa provided parents with alternative and proactive strategies to manage their children’s misbehavior to forestall the use of harsher forms of discipline. Facilitators were trained to model and reinforce these alternative strategies, elicit their benefits in discussions with parents, and minimize the attention or time given to responses that endorse corporal punishment.

### Phases 3 and 4: Preliminary Testing and Refinement of the Adapted MaPa Program

Following the aforementioned adaptations, the adapted MaPa program was implemented in a feasibility trial. Qualitative FGDs with 19 parents and three facilitators were conducted after the trial to explore participants’ experiences and perceptions about MaPa program content and delivery. The themes from the FGDs presented here pertain to the contextual acceptability and relevance of the program and present implications for its further refinement.

#### Describing actions and feelings as challenging practices

Parents shared that they rarely described actions and feelings with their children since it was difficult and awkward. MaPa facilitators commented that this behavior, as well as communicating about emotions, seemed unnatural for most parents as they typically do not interact with their children in highly verbal ways or articulate their children’s feelings. As a parent said:

Honestly, I’m not used to doing it…I see my son watching television and I know that he is happy. I don’t have to say, ‘Son, you are so happy’.

Parents were especially less likely to comment on or label their children’s negative emotions because they believed that doing so would intensify the child’s emotion and its expression. This observation is consistent with studies indicating that direct parent engagement with children and child‐directed speech is less frequent among families of lower socioeconomic status (Hoff, 2003).

Despite challenges experienced by parents in applying these practices, we retained content on describing actions and feelings in the MaPa program, in keeping with compelling scientific evidence that highlights their benefits for children’s cognition and language (Rowe, 2012) and emotional competence (Denham, Renwik‐DeBardi, & Hewes, 1994). Even in cultures where adult engagement with children is not normative, parents’ use of child‐directed speech and, correspondingly, children’s vocabulary increased when interventions included these components (Weber, Fernald & Diop, 2017). We improved the session materials to clarify the objectives of these practices to parents and more clearly elicit their benefits (e.g., why we should enhance children’s language skills). Describing actions and feelings were framed as activities that parents could do more naturally during their one‐on‐one time with their children and could focus on the environment such as naming the actions or feelings of other people and commenting on objects and events that children could observe.

#### Meaningful program content

Parents understood the meaning underlying the metaphor of the *Masayang Tahanan* (“We must strengthen the walls, otherwise the roof might fall”) and underscored how it was evident in their experiences (“I have not used cool down and consequences on my daughter because she has really behaved well, and we have become closer to each other”). The parents also spoke about a gradual transformation in attitudes (“I thought that I needed to be obeyed at all times. I realized that I also needed to consider my son’s preferences and acknowledge his choices”) and behavior (“Spanking was part of how I was raised. I do not have to apply it to my son.”).

Parents also perceived that praise and rewards, positive instructions, and household rules helped them to achieve their goals of having children who exhibit *mabuting asal* (good behavior). Even if praise and rewards may not be their typical approach to supporting children, Filipino parents gave positive feedback on this aspect of program content. Parents also found the use of positive instructions and household rules helpful and effective in strengthening children’s compliance.

Finally, parents found the mindfulness‐based stress‐reduction techniques helpful in regulating their anger and impulse to hit their children or argue with their spouses. One parent emphasized this point by saying “I became conscious of my temper when I joined MaPa. I used to spank or scream at my children when they misbehaved. Nowadays, I speak calmly and with affection when I talk to them.” The parenting intervention’s impact on the entire family system is underscored by the statement of one mother: “When my child and I no longer fight often, my husband and I also no longer fight often.”

#### Effective MaPa delivery approaches

Parents shared that they liked the *komiks* as these depicted scenarios and challenges that were typical of Filipino family life. Parents also reported that the collaborative approach and role‐plays were helpful in their learning of skills and that they preferred these methods to lectures. As a central part of both the original and adapted programs, parents were treated as experts in parenting their children, enhancing feelings of respect and program ownership. Mothers reported feeling pride and confidence as parents, and becoming more active and assertive in intervening with the spouse and other household caregivers on matters of childrearing. However, they also shared that they had challenges with the assigned home activities due to other responsibilities such as work and domestic chores.

Parents appreciated the text message reminders that they received from their facilitators as these made them feel welcome and valued. Although the home visits made some parents feel embarrassed, they shared that these visits also encouraged them to attend sessions and enhanced their appreciation of their facilitators, whom they perceived as providing personalized attention. Such interactions between facilitators and participants may be especially important in the Filipino culture which promotes *pakikipagkapwa*, or valuing and respecting the other regardless of status differences.

## General Discussion

This paper addresses the need to document program adaptation procedures in a systematic manner (e.g., Mejia et al., 2016) and underscores the benefits of conducting a multistage process to inform the South‐to‐South cultural adaptation of an evidence‐based parenting program for vulnerable families in the Philippines (Barrera et al., 2013). Parent data, local research, and scientific evidence informed our adaptation process.

We note that the insider or emic perspective provided by the Filipino researchers was a critical resource in the process and highlights the importance of collaboration with the end users and providers of the program. Because the project involved international collaborators (with backgrounds from the United States, United Kingdom, and South Africa), the cultural adaptation process required balancing the points of view of cultural insiders and outsiders. Disagreements presented opportunities to learn about each other’s assumptions, perspectives, and experiences. For example, using food (e.g., a favorite dish) as a reward is meaningful in the Philippine context, where offering food is a significant gesture of hospitality and signals deepening relationships. However, a Western perspective might consider food to be inappropriate as a reinforcer given unhealthy eating habits or problems such as obesity. Geographic distance and the need to communicate via online channels likewise complicated project management and sometimes made it difficult to immediately appreciate local ways and constraints in achieving project goals. Nevertheless, the international partners gave the insiders’ interpretations significant weight and supported the research and adaptation process to the fullest extent.

Our participatory approach empowered Filipino parents as active agents in the design of an intervention that will ultimately be delivered widely in their context. The Filipino researchers themselves can be considered outsiders in the specific environment of the target families, where deprivation and adversity are prevalent. Hence, it was critical to include and respect the values, insights, and preferences of local informants, and acknowledge the heterogeneity of knowledge and experiences even within one sociocultural setting. Cross‐ and within‐culture program transportation and adaptation are increasingly important to study, given heightened opportunities for international collaboration and global initiatives promoting social development in LMICs. We found that continuous dialogue, reflexivity, openness, and adaptability among collaborators and stakeholders were key ingredients in the process of transporting and culturally adapting intervention programs.

With respect to the MaPa program, we largely retained the PLH‐YC theory of change, content, and delivery approach. However, we grounded the adapted program in prevailing Filipino family values and attitudes that corresponded with the needs identified by Filipino parents. Our adaptation therefore addressed both surface and deep structures (Resnicow et al., 2000), in that all of the content was nuanced by Filipino values while we maintained the original principles in content and delivery.

We regarded MaPa content that parents found challenging or awkward as opportunities to reflect on the core values of the recipients and to refine program messages to be more in line with these values. For instance, given the research describing the tendency of Filipino parents to subscribe to authoritarian attitudes (Alampay & Jocson, 2011; Jocson et al., 2012), we carefully worded our messages around child‐directed play so as to make the activity more acceptable for parents. Instead of pressuring them to change their attitudes or behavior (e.g., corporal punishment), we supported emerging new skills that are consonant with Filipino values. We acknowledged that verbally engaging with children (i.e., describing actions and feelings) was new and challenging for low‐income Filipino parents, and so provided additional support for the practice given its benefits for children’s development.

Reinforcing the cultural value of *pakikipagkapwa* or treating the other as equal, facilitators adopted a supportive, nonjudgmental stance toward the program recipients in order to support parents’ efficacy and confidence in relating with their children, spouses, and other household caregivers. Beyond effects on parenting and child maltreatment, the potential of parenting interventions to empower caregivers in disadvantaged and abusive circumstances can be systematically evaluated in formal trials.

Our work is consistent with evidence that numerous parenting goals and values are comparable across cultures and contexts, and therefore warrant similar support systems or intervention content (Leijten et al., 2016). The program elements that Filipino parents identified as necessary are among those that are commonly targeted by evidence‐based parenting interventions. Moreover, intervention principles, such as the enhancement of parent–child relationships through positive attention, and modification of parent and child behavior via social learning strategies, have been found to remain effective across different contexts (Gardner et al., 2016; Leijten, et al., 2018; Leijten et al., 2016). While this underscores the transportability of interventions, systematic adaptation as described in this paper enhances program acceptability and cultural relevance and sensitivity in specific settings. The top‐down plus bottom‐up adaptation and collaborative approach and principles of dialogue and reflexivity are applicable to program developers and service providers engaging in family interventions, including family therapists and social workers in clinical, school, or community settings. However, evidence of program effectiveness vis‐à‐vis adapted components remains critical, or intervention efforts are meaningless.

More broadly, the work of adapting intervention programs in low‐resource contexts should recognize the wider systemic inequities and oppressive norms (e.g., gender) that are associated with higher rates of violence in communities and families. The felt pressures of economic deprivation, such as those experienced by the families in our Filipino sample, have been shown to exacerbate familial stress, mental health problems, marital conflict and intimate partner violence, and harsh parenting behaviors (Conger, Conger & Martin, 2010). That the MaPa program is to be embedded in a social welfare delivery system (i.e., CCT) recognizes that parenting support interventions need to be inextricably linked with social development programs to alleviate poverty, enhance caregiver and child health and education, and support gender equality. Such an integrated strategy is critical in LMICs, where cost‐effectiveness and scalability of programs are particular concerns and challenges (Ward et al., 2015). Beyond the process of culturally adapting and transporting a program, future research should document processes and good practices from program adaptation to program scale‐up and integration with broader social system initiatives to prevent violence in disadvantaged families.

A number of limitations qualify our conclusions. We collected data from Filipino parents in a single low‐resource urban community, and their needs may not reflect low‐income Filipino families living in other settings. We focused on qualitative methods in our cultural adaptation of PLH‐YC for the Philippines. Future studies on cultural adaptation of parenting programs should integrate both qualitative and quantitative methods to provide a more multifaceted investigation of the adaptation process and program impact, providing a richer analysis of feasibility, acceptability, and effectiveness. Further testing of intervention effectiveness in an RCT is necessary to confirm whether the culturally adapted MaPa program is effective in achieving its aims to reduce child maltreatment and improve child and parent well‐being for low‐income Filipino families.

## Supporting information


**Figure S1A.** The Rondavel of Support and Masayang Tahanan (Happy Home).

**Figure S1B.** The Rondavel of Support and Masayang Tahanan (Happy Home).

**Figure S2A.** Sample Comics on One‐on‐One Time from PLH for Young Children (top) and MaPa Program (bottom).

**Figure S2B.** Sample Comics on One‐on‐One Time from PLH for Young Children (top) and MaPa Program (bottom).

## Footnotes

^1^

https://www.who.int/violence_injury_prevention/violence/child/plh/en

^2^
The difference in target age ranges for the original and adapted program was driven by donor constraints. The age range covers the highest risk group for children to experience violence based on the Violence Against Children Study (i.e., 4–5 years old).
