# Supplementary figures and images for: A South‐to‐South Cultural Adaptation of an Evidence‐Based Parenting Program for Families in the Philippines
Source: Fam Process. 2021 Jan 6;60(4):1202–16. doi: 10.1111/famp.12625 (PMC9189706; doi:10.1111/famp.12625)

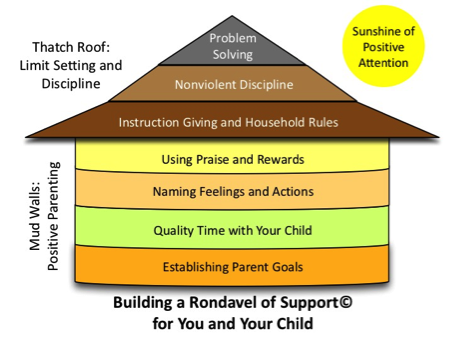

Supplement: Supplementary file 1 — Figure S1A. The Rondavel of Support and Masayang Tahanan (Happy Home). [file FAMP-60-1202-s002.png]

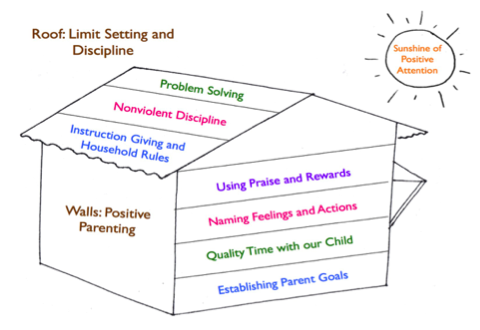

Supplement: Supplementary file 2 — Figure S1B. The Rondavel of Support and Masayang Tahanan (Happy Home). [file FAMP-60-1202-s001.png]

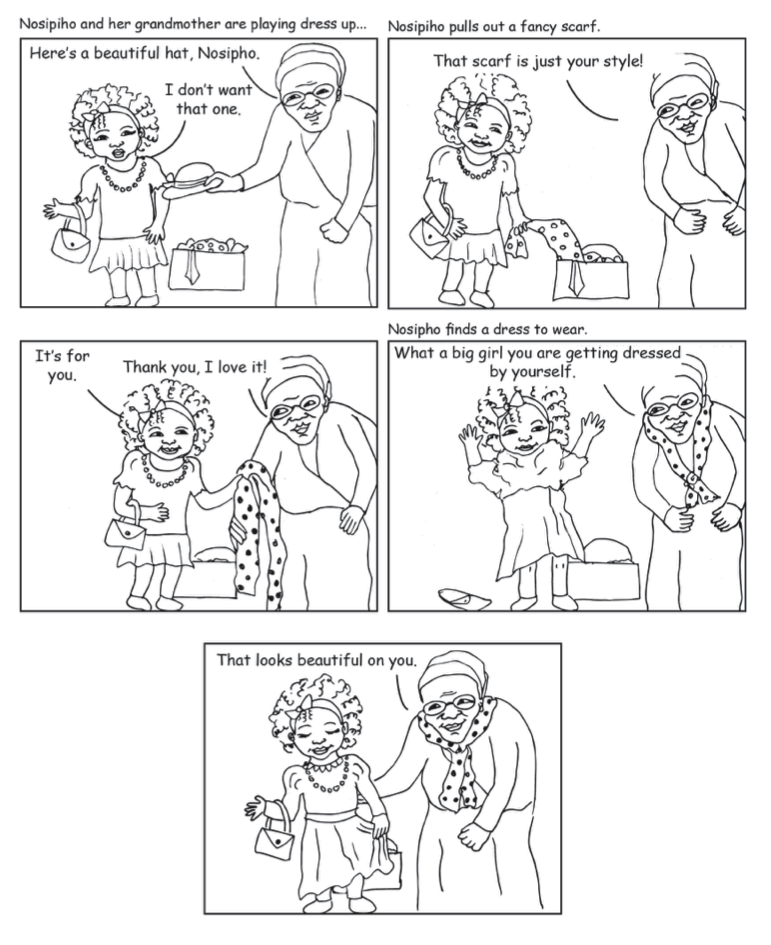

Supplement: Supplementary file 3 — Figure S2A. Sample Comics on One‐on‐One Time from PLH for Young Children (top) and MaPa Program (bottom). [file FAMP-60-1202-s004.png]

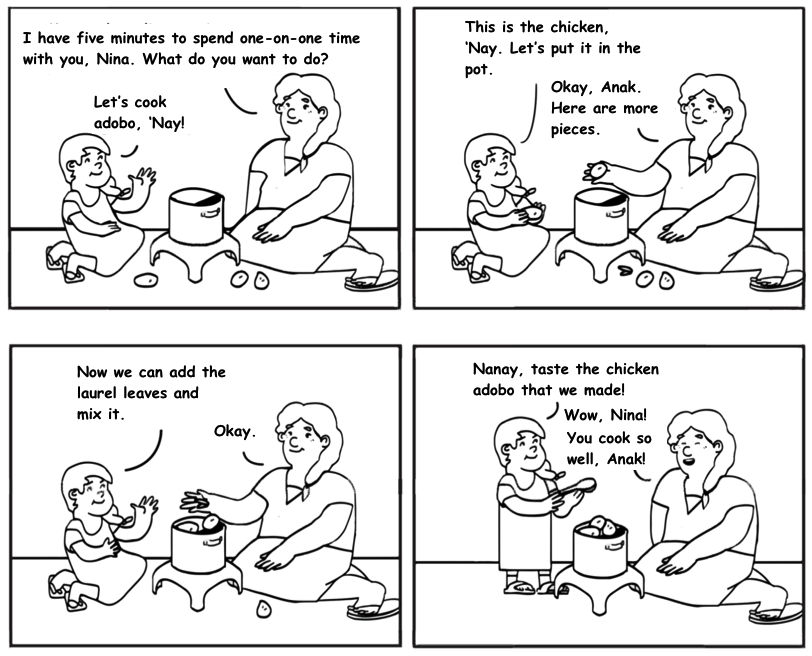

Supplement: Supplementary file 4 — Figure S2B. Sample Comics on One‐on‐One Time from PLH for Young Children (top) and MaPa Program (bottom). [file FAMP-60-1202-s003.png]
